# Diagnostic Significance of Circulating EBV-miR-BHRF1-3 and EBV-miR-BART2-5p in Lymphoma A Case-Control Study

**DOI:** 10.12669/pjms.42.4.14739

**Published:** 2026-04

**Authors:** Asli Kara, Murat Kizilkaya, Eda Balkan, Fuat Erdem, Hasan Turkez

**Affiliations:** 1 Asli Kara Department of Internal Medicine, Faculty of Medicine, Atatürk University, Erzurum, Türkiye; 2 Murat Kizilkaya, Department of Medical Biology, Faculty of Medicine, Ağrı İbrahim Çeçen University, Ağrı, Türkiye; 3 Eda Balkan, Department of Medical Biology, Faculty of Medicine, Atatürk University, Erzurum, Türkiye; 4 Fuat Erdem, Department of Internal Medicine, Faculty of Medicine, Atatürk University, Erzurum, Türkiye; 5 Hasan Türkez, Department of Medical Biology, Faculty of Medicine, Atatürk University, Erzurum, Türkiye

**Keywords:** Circulating biomarkers, EBV, lymphoma, miRNA, qRT-PCR

## Abstract

**Objective:**

Epstein-Barr virus (EBV)-derived microRNAs (miRNAs) have been proposed as potential circulating biomarkers in EBV-associated malignancies. This study aimed to quantify the expression levels of four EBV-miRNAs (ebv-miR-BHRF1-2-5p, BHRF1-3, BART1-3p, and BART2-5p) in peripheral blood and to explore their possible diagnostic value in distinguishing lymphoma patients from healthy individuals.

**Methodology:**

This case control study included 50 newly diagnosed lymphoma patients (22 Hodgkin lymphoma, 28 Non-Hodgkin lymphoma) and 50 healthy controls recruited in 2025 at the Department of Internal Medicine (Hematology), Atatürk University. qRT-PCR analyses were performed at the Department of Medical Biology. EBV-miRNA expression levels were calculated using the ΔCt method with U6 snRNA as an internal control. Statistical analyses included FDR correction and ROC curve evaluation.

**Results:**

Ct values for U6 snRNA and all four EBV-miRNAs were significantly lower in lymphoma patients compared with controls (all p < 0.001, FDR < 0.001), indicating increased levels of circulating viral transcripts. ROC analyses showed that some miRNAs, particularly BHRF1-3 and BART1-3p, demonstrated notable discriminatory ability between patients and controls. No significant overall differences were observed between Hodgkin and Non-Hodgkin lymphoma; however, subtype analysis revealed significant variation in BHRF1-3 and BART2-5p expression (FDR = 0.005).

**Conclusion:**

Circulating EBV-miRNAs were elevated in lymphoma patients, and several miRNAs showed promising discriminatory performance. However, this study is limited by its single-center design and the absence of external validation. Therefore, these findings should be considered preliminary, and further research in larger and independent cohorts is needed to confirm the diagnostic utility of EBV-derived miRNAs in lymphoma.

## INTRODUCTION

Epstein-Barr virus (EBV) is a ubiquitous herpesvirus implicated in the pathogenesis of numerous lymphoproliferative malignancies.^1^,^2^ EBV infection is detected in approximately 30-50% of classical Hodgkin lymphoma (HL) cases and in selected subtypes of non-Hodgkin lymphoma (NHL), particularly in endemic Burkitt lymphoma and extranodal NK/T-cell lymphoma.^3^-^7^ MicroRNAs (miRNAs) are small non-coding RNA molecules that regulate gene expression at the post-transcriptional level by binding to target mRNAs. 8-12 During latent infection, EBV expresses a restricted set of viral genes (including EBNA1, latent membrane proteins, and EBERs) as well as two major clusters of viral microRNAs-BHRF1 and BART miRNAs.^8^ These viral miRNAs are abundantly expressed in EBV-positive tumor cells and have emerged as promising biomarker candidates for EBV-associated cancers.^5^,^13^

Circulating microRNAs represent an attractive source of non-invasive biomarkers due to their stability in blood samples. Several studies have demonstrated elevated levels of viral miRNAs in the serum or plasma of patients with EBV-associated malignancies. For example, Komabayashi et al. reported markedly increased expression of multiple BART miRNAs in patients with nasal NK/T-cell lymphoma compared with healthy individuals.^14^ Moreover, elevated levels of EBV-miR-BHRF1-1 observed even in chronic lymphocytic leukemia (CLL) cases-traditionally not considered EBV-associated-suggest that viral miRNAs may serve as broader indicators of lymphoproliferative activity.^15^

EBV-derived miRNAs contribute to oncogenic processes by modulating host immune responses and regulating viral gene expression, thereby facilitating immune evasion and dampening inflammatory signaling. BHRF1-2-5p, for instance, targets IL-1R1 to attenuate inflammatory activation,^16^,^17^ while BHRF1-3 suppresses CXCL11, potentially enabling escape from immune surveillance.^18^

In this study, we compared circulating levels of BHRF1-2-5p, BHRF1-3, BART1-3p, and BART2-5p between lymphoma patients and healthy controls, and evaluated their diagnostic performance as well as their associations with clinical characteristics.

## METHODOLOGY

This case-control study was conducted in 2025 at the Departments of Internal Medicine (Hematology) and Medical Biology, Atatürk University. The study included 50 newly diagnosed lymphoma patients and 50 healthy controls. Lymphoma diagnoses were confirmed by histopathology and immunophenotyping according to WHO criteria. Control subjects had no history of malignancy or acute infection. Individuals with active EBV infection, HIV positivity, or immunosuppressive therapy were excluded.

### Ethical approval:

It was granted by the Ataturk University Faculty of Medicine Ethics Committee (Approval No: B56.6 ATA-0.01.00/6-56:2025) and written informed consent was obtained from all participants.

### Sample Collection and RNA Extraction:

Peripheral blood (5 mL) was collected before treatment. Plasma was separated and stored at -80°C. Total RNA, including small RNAs, was isolated from 200 μL plasma using a commercial miRNA extraction kit following the manufacturer’s protocol.

### cDNA Synthesis and qRT-PCR:

Extracted RNA was reverse transcribed using stem-loop primers specific for the four EBV miRNAs and the endogenous control U6 snRNA. Quantitative PCR was performed using a real-time PCR system (SYBR Green chemistry). All reactions were run in duplicate, and no-template controls were included.

### miRNA Quantification:

Relative expression was calculated using the ΔCt method (Ct_miRNA - Ct_U6). Lower ΔCt values indicate higher expression. Because miRNA Ct values were non-normally distributed, medians and ranges were reported.

### Statistical analysis:

Data were analyzed using IBM SPSS version 23. The normality of distribution was assessed with the Kolmogorov-Smirnov and Shapiro-Wilk tests. Associations between groups and categorical variables were examined using Yates’ continuity correction. For group comparisons, the Independent Samples t-test was applied to normally distributed variables, whereas the Mann-Whitney U test was used for non-normally distributed variables. In comparisons involving three or more groups, one-way ANOVA was used for normally distributed variables and the Kruskal-Wallis test for non-normally distributed variables. Relationships between non-normally distributed variables were evaluated using Spearman’s rho correlation coefficient. ROC analysis was performed to determine optimal cut-off values for variables that discriminated the patient group from controls. Results were presented as frequency (percentage) for categorical variables and as mean ± standard deviation or median (minimum-maximum) for quantitative variables. A significance level of p < 0.050 was considered statistically significant.

## RESULTS

A total of one hundred participants were enrolled in the study, consisting of 50 lymphoma patients and 50 healthy controls. The distribution of sex was comparable between groups (p = 1.000); however, the mean age was significantly higher in the patient group (43.1 ± 15.02 vs. 36.53 ± 8.79 years; p = 0.011). Among lymphoma cases, 44% were diagnosed with Hodgkin lymphoma (HL) and 56% with non-Hodgkin lymphoma (NHL), with EBV positivity detected in 54% of patients. Subtype analysis showed that diffuse large B-cell lymphoma accounted for 27.1% of cases, follicular lymphoma for 22.9%, and the nodular sclerosis and mixed cellularity variants of classical HL for 27.1% and 18.8%, respectively. Most patients presented with advanced disease, with 62% classified as stage III-IV. B symptoms were present in 24% of cases, while bulky disease was observed in 16.2%.

**Fig.1:**
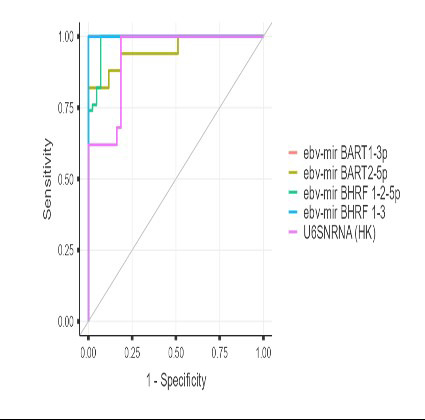
Receiver operating characteristic (ROC) curves of EBV-encoded microRNAs and U6 snRNA for distinguishing lymphoma patients from healthy.

Immunohistochemical evaluation demonstrated a predominant B-cell phenotype, reflected by CD20 positivity in 42.6% and PAX5 positivity or partial positivity in 63.9% of cases, whereas CD3 expression was limited (14.3%). CD30 expression was detected in approximately half of the patients (51.2%). BCL6 (83.3%) and BCL2 (60%) positivity rates were high, and MUM1 positivity (70%) supported an activation-associated immunophenotype. CD32 expression was heterogeneous, being positive in one-third of cases. ALK and CD2 were negative in all samples, while EMA positivity was limited (33.3%). CD15 and CD10, characteristic markers for HL, were positive in all evaluable samples. Proliferative activity was high, with a mean Ki-67 index of 58.29% (median 60%; range 30-100%), and C-MYC expression averaged 25% (range 20-30%).

All EBV-encoded miRNAs were markedly elevated in lymphoma patients. Ct values for U6 snRNA and all four EBV-miRNAs were significantly lower in the patient group compared with healthy controls (p < 0.001, FDR < 0.001 for all variables). In qRT-PCR methodology, this reduction in Ct values indicates higher transcript abundance. The median Ct of U6 decreased from 24.66 in controls to 20.25 in patients, reflecting an overall increase in small RNA load. Similarly, significant decreases in Ct values for BHRF1-2-5p (27.21 → 20.50), BHRF1-3 (25.15 → 20.08), BART1-3p (27.75 → 20.31), and BART2-5p (28.63 → 20.99) demonstrated robust elevations of all viral miRNAs in the patient group. Each of the measured EBV-miRNAs distinguished patients from controls with strong statistical significance (p < 0.001). These findings confirm that circulating EBV-miRNA expression is markedly increased in lymphoma and support their potential as diagnostic biomarkers (Table-I).

**Table-I:** Differential Expression of Circulating EBV MicroRNAs Between Lymphoma Cases and Controls.

	Groups		Total	Test Statistic	p^x^	FDR- corrected p
	Control	Patient				
U6SNRNA (HK)	24,66 (21,11: 26,69)	2,25 (16,77: 22,12)	21,8 (16,77: 26,69)	149,000	<0,001	<0,001
ebv-mir BHRF 1-2-5p	27,21 (22,19: 32,9)	20,5 (14,56: 24,89)	23,68 (14,56: 32,9)	34,000	<0,001	<0,001
ebv-mir BHRF 1-3	25,15 (23,35: 32,21)	20,08 (13,47: 23,29)	22,84 (13,47: 32,21)	0,000	<0,001	<0,001
ebv-mir BART1-3p	27,75 (23,34: 32,9)	20,31 (18,9: 21,91)	21,54 (18,9: 32,9)	0,000	<0,001	<0,001
ebv-mir BART2-5p	28,63 (25,15: 32,95)	20,99 (15,83: 28,91)	25,81 (15,83: 32,95)	105,000	<0,001	<0,001

When Hodgkin and Non-Hodgkin lymphoma groups were compared, no significant differences were observed in the Ct values of U6 snRNA or any of the four EBV-encoded miRNAs (all p > 0.20, FDR-adjusted p > 0.39). Both HL and NHL patients exhibited comparable Ct ranges, indicating that circulating viral miRNA expression increases irrespective of lymphoma subtype. This suggests that EBV-derived miRNAs primarily reflect the presence of lymphoma and systemic EBV activity rather than specific tumor subtypes. The absence of significant group separation supports the notion that viral miRNA levels rise to a similar extent in HL and NHL through shared underlying biological mechanisms.

When lymphoma subtypes were further evaluated, no significant differences were detected in U6 snRNA or most EBV-encoded miRNAs (all p > 0.05, FDR > 0.05). However, EBV-miR-BHRF1-3 levels differed significantly among subtypes (p = 0.018, FDR = 0.005). Likewise, EBV-miR-BART2-5p demonstrated statistically significant variation across subtypes (p = 0.044, FDR = 0.005). In contrast, EBV-miR-BHRF1-2-5p and EBV-miR-BART1-3p did not show meaningful variation between groups (all p > 0.05, FDR > 0.05). These findings indicate that some viral miRNAs exhibit subtype-specific Ct distributions among lymphoma categories (Table-II).

**Table-II:** Comparison of Circulating EBV-encoded microRNA Levels Between Hodgkin and Non-Hodgkin Lymphoma.

		U6SNRNA (HK)	ebv-mir BHRF 1-2-5p	ebv-mir BHRF 1-2-5p	ebv-mir BHRF 1-2-5p	ebv-mir BART2-5p
Diagnosis	Hodgkin	20.17 (16.77: 21.82)	20.78 (14.56: 24.89)	35.44 (13.47: 23.29)	20.24 ± 0.72	21.13 (15.83: 28.91)
	Non-Hodgkin	20.58 (16.77: 22.12)	20.5 (14.56: 24.89)	20.28 (13.47: 23.29	20.41 ± 0.92	30.99 (15.83: 28.91)
	Test Statistic	244.000	250.000	243.000	-0.750	297.000
	p	0.210^x^	0.256^x^	0.020^x^	0.457^y^	0.029^x^
	FDR-Corrected p	0.394	0.427	0.005	0.685	0.005
SUBTYPE	Diffuse Large B-Cell	21.7 (17.7: 22.04	20.5 (14.96: 23.68)	20.45 (13.47: 22.84)	20.62 (18.9: 21.91	21.54 (15.83: 28.91)
	Follicular	19.89 (16.77: 22.04)	20.37 (14.56: 24.89)	20.11 (14.57: 23.29)	20.2 (19.27: 21.91	20.99 (15.83: 28.91)
	Classical Hodgkin	19.29 (16.77: 21.8)	21.34 (20.78: 21.9)	18.49 (18.15: 18.82)	20.49 (20.44: 20.54)	23.18 (19.52: 26.84)
	Classical Hodgkin - Mixed Cellularity	20.25 (17.7: 21.82)	20.37 (16.94: 24.89)	36.72 (13.47: 32.36)	20.2 (18.9: 21.54)	20.99 (19.52: 28.91)
	Classical Hodgkin - Nodular Sclerosis	20.25 (16.77: 22.12	21.26 (14.56: 23.75)	20.11 (14.57: 23.29)	20.44 (19.27: 21.17)	28.43 (15.83: 26.84)
	Test Statistic	1.888	1.401	7.591	1.278	3.085
	P^x^	0.756	0.844	0.018	0.865	0.044
	FDR-Corrected p	0.865	0.865	0.005	0.865	0.005

## DISCUSSION

In this case-control study, we demonstrated that all four EBV-derived microRNAs (BHRF1-2-5p, BHRF1-3, BART1-3p, and BART2-5p) were markedly elevated in the peripheral blood of lymphoma patients compared with healthy individuals. The significantly reduced Ct values across all viral miRNAs (all p < 0.001) indicate a substantial increase in circulating viral transcript abundance, confirming a strong systemic EBV-associated molecular signature. The diagnostic performance was particularly striking: ROC analyses revealed exceptionally high accuracy, with BHRF1-3 and BART1-3p achieving 100% sensitivity and specificity. These findings highlight the potential of EBV-miRNAs as powerful, minimally invasive biomarkers capable of distinguishing lymphoma patients from healthy individuals with remarkable precision, consistent with previous studies in EBV-associated malignancies.^14^-^15^

When Hodgkin lymphoma (HL) and Non-Hodgkin lymphoma (NHL) were compared, no significant differences were observed in viral miRNA levels, suggesting that EBV-miRNA upregulation reflects a shared biological process independent of classical lymphoma categorization, consistent with previous reports describing EBV-miRNAs as regulators of EBV-associated lymphomagenesis.^19^ This pattern is consistent with prior studies reporting that EBV-miRNAs act as broad molecular indicators of latent EBV activity, immune dysregulation, and virus-driven oncogenic pathways across multiple EBV-associated malignancies.^19^ Such observations align with data showing that EBV-miRNAs regulate interferon responses, alter cytokine networks, and reshape the tumor microenvironment.^13^

However, detailed subtype analysis revealed that two miRNAs-BHRF1-3 and BART2-5p-showed significant variation across HL and NHL subgroups (FDR = 0.005). Notably, the nodular sclerosis and mixed cellularity subtypes of classical HL exhibited distinct expression patterns. These findings align with previous reports suggesting that these HL variants harbor higher EBV burdens and demonstrate more active EBV-related transcriptional programs. Mechanistically, BHRF1-3 is implicated in suppressing CXCL11 and diminishing T-cell recruitment, whereas BART2-5p is known to interfere with antigen presentation-mechanisms that collectively contribute to viral immune evasion.^18^,^20^ Thus, the subtype-specific differences observed in our study may reflect biologically relevant EBV-tumor interactions driven by viral miRNA-mediated modulation of host immunity.

**Table-III:** Comparison of quantitative variables according to categorical variables.

Variable/Category		U6 snRNA	BHRF1-2-5p	BHRF1-3	BART1-3p	BART2-5p
Ki-67 (r)		0.280	-0.142	0.208	0.046	-0.013
Ki-67 (p)		0.088	0.393	0.211	0.078	0.937
Ki-67 (FDR)		0.330	0.627	0.527	0.330	0.937
CD20 - Negative		20.25-21.82	20.3±3.18	19.47±2.61	20.35±0.7	21.42±3.26
CD20 - Positive		19.89-21.8	19.28±3.97	20.26±3.09	20.02±0.72	20.56±1.37
	Test Statistic	2,563	0,253	0,681	1,071	0,765
	p	0,464^x^	0,859^y^	0,568^y^	0,372^y^	0,520^y^
	FDR-Corrected p	0,633	0,920	0,655	0,627	0,650
PAX5 - Negative		21.8-22.12	22.37±2.82	17.44±3.0	20.32±0.54	20.43±2.84
PAX5 - Positive		20.17-22.04	20.37±3.75	20.08±2.0	20.58±1.91	21.41±2.28
	Test Statistic	4,018	9,384	11,64	6,161	3,911
	p	0,404^x^	0,052^x^	0,020^x^	0,187^x^	0,418^x^
	FDR-Corrected p	0,627	0,330	0,300	0,527	0,627
BCL6 - Negative		20.75-21.71	20.44±3.2	21.74±2.84	20.25±1.16	20.45±2.0
BCL6 - Positive		21-22.04	20.25±3.2	20.54±2.89	20.31±1.21	21.51±2.81
	Test Statistic	7,000	10,000	5,000	8,000	6,000
	p	0,519^z^	1,000^z^	0,283^z^	0,667^z^	0,390^z^
	FDR-Corrected p	0,838	1,000	0,838	0,838	0,838
BCL2 - Negative		20.25-22.12	20.56±1.94	18.61±2.95	20.41±0.73	20.99±2.48
BCL2 - Positive		20.08-22.04	19.71±3.23	19.76±2.68	20.4±1	21.47±3.81
	Test Statistic	0,841	0,833	1,157	0,071	0,615
	p	0,657^x^	0,444^y^	0,327^y^	0,932^y^	0,735^x^
	FDR-Corrected p	0,838	0,838	0,838	0,999	0,848
CD32 - Negative		20.3±1.68	20.44±3.68	19.74±2.56	20.52±0.85	22.73±2.82
CD32 - Positive		20.47±1.37	23.68±4.81	18.78±2.65	20.27±0.65	20.63±2.19
	Test Statistic	0,677	1,692	0,407	0,406	2,188
	p	0,516^y^	0,429^x^	0,669^y^	0,670^y^	0,145^y^
	FDR-Corrected p	0,677	1,692	0,407	0,406	2,188

Importantly, no meaningful associations were found between EBV-miRNA levels and immunohistochemical markers (including CD20, PAX5, BCL6, BCL2, MUM1, CD30), nor with proliferative activity assessed by Ki-67. This suggests that EBV-miRNA elevation is independent of tumor phenotype or proliferation index and may instead represent an EBV-specific systemic process. Such independence underscores the value of EBV-miRNA profiling as a complementary tool alongside traditional histopathology and immunophenotyping.

### Strength of the Study:

This study provides preliminary findings demonstrating that circulating EBV-derived microRNA levels can be evaluated in patients with lymphoma. A strength of the study is the analysis of EBV-miRNA expression using real-time PCR within a case-control design. The findings suggest that EBV-miRNA profiling may contribute to a better understanding of lymphoma biology.

### Limitations:

It includes the relatively small sample size and single-center design may limit generalizability. In addition, long-term follow-up data and functional validation analyses were not available. Larger multicenter studies are needed to confirm these findings.

## CONCLUSION

Taken together, our findings indicate that circulating EBV-miRNAs are robust and reliable biomarkers for lymphoma detection, capable of discriminating patients from healthy controls with excellent accuracy. While EBV-miRNA elevation appears to be a global feature of lymphoma, subtype-specific patterns observed for BHRF1-3 and BART2-5p may offer additional insight into biological heterogeneity and could potentially contribute to diagnostic refinement or prognostic assessment. Larger multicenter studies integrating EBV-miRNA profiling with viral load measures, cytokine signatures, and host immune markers will further elucidate their clinical utility in diagnosis, risk stratification, and treatment response monitoring.
